# Risk factor profiles and clinical outcomes for children and adults with pneumococcal infections in Singapore: A need to expand vaccination policy?

**DOI:** 10.1371/journal.pone.0220951

**Published:** 2019-10-16

**Authors:** Rosario Martinez-Vega, Elita Jauneikaite, Koh Cheng Thoon, Hui Ying Chua, Amanda Huishi Chua, Wei Xin Khong, Ban Hock Tan, Jenny Low Guek Hong, Indumathi Venkatachalam, Paul Anantharajah Tambyah, Martin L. Hibberd, Stuart C. Clarke, Oon Tek Ng

**Affiliations:** 1 Department of Infectious Diseases, Tan Tock Seng Hospital, Singapore, Singapore; 2 Infectious Diseases, Genome Institute of Singapore, Singapore, Singapore; 3 Faculty of Medicine and Institute of Life Sciences, University of Southampton, Southampton, United Kingdom; 4 Infectious Disease Service, Department of Paediatrics, KK Women’s and Children’s Hospital, Singapore, Singapore; 5 Duke-NUS Graduate Medical School, Singapore, Singapore; 6 Department of Infectious Diseases, Singapore General Hospital, Singapore, Singapore; 7 Division of Infectious Diseases, National University of Singapore, Singapore, Singapore; 8 National Centre for Infectious Diseases (NCID), Singapore, Singapore; 9 Lee Kong Chian School of Medicine, Nanyang Technological University, Singapore, Singapore; Universidade de Lisboa Faculdade de Medicina, PORTUGAL

## Abstract

Invasive pneumococcal infection is a major cause of morbidity and mortality worldwide despite the availability of pneumococcal vaccines. The aim of this study was to re-evaluate the clinical syndromes, prognostic factors and outcomes for pneumococcal disease in adults and children in Singapore during the period before and after the introduction of the pneumococcal vaccine. We retrospectively analyzed a large cohort of patients admitted to the four main public hospitals in Singapore with *S*. *pneumoniae* infection between 1997 and 2013. A total of 889 (64% of all isolates identified in the clinical laboratories) cases were included in the analysis; 561 (63.1%) were adult (≥16 years) cases with a median age of 62 years and 328 (36.9%) were paediatric cases with a median age of 3 years. Bacteraemic pneumonia was the most common syndrome in both groups (69.3% vs. 44.2%), followed by primary bacteraemia without pneumonia (14.3% vs. 13.4%), meningitis (6.4% vs. 7.6%) and non-bacteraemic pneumonia (5.2% vs. 21%). The major serotypes in adults were 3, 4, 6B, 14, 19F and 23F whereas in children they were 14, 6B and 19F, accounting both for nearly half of pneumococcal disease cases. No particular serotype was associated with mortality or severity of the pneumococcal disease. Overall mortality rate was 18.5% in adults and 3% in children. Risk factors for mortality included acute cardiac events in adults, meningitis in children and critical illness and bilateral pulmonary infiltrates in both adults and children. Penicillin resistance was not associated with increased mortality. Our results agree with global reports that the course of pneumococcal disease and its clinical outcome were more severe in adults than in children. The main serotypes causing invasive disease were mostly covered by the vaccines in use. The high mortality rates reflect an urgent need to increase vaccination coverage in both adults and children to tackle this vaccine-preventable infection.

## Introduction

*Streptococcus pneumoniae* (pneumococcus) is one of the most common causes of morbidity and mortality among children and adults worldwide, with an estimated 800,000 annual deaths in children under 5 years of age globally [1]. Though colonization with pneumococci is mostly asymptomatic, it can cause a range of diseases from mild localized infections such as acute otitis media and sinusitis, to more severe infections such as pneumonia, bacteraemia and meningitis [2].

Pneumonia with empyema and/or bacteraemia, febrile bacteraemia and meningitis are the most common manifestations of invasive pneumococcal disease (IPD). While children under the age of 2 years and adults over 65 years of age are reportedly at higher risk of IPD [3], comorbidities such as diabetes mellitus, chronic heart and lung conditions, and immune deficiencies also increase the risk of developing pneumococcal disease [3–5].

The ability of pneumococcus to cause disease is directly related to the production of polysaccharide capsule, a structure that provides resistance to phagocytosis and allows host immune evasion by the bacteria [6]. To date, 98 antigenically distinct capsules have been reported in pneumococcus, corresponding to 98 distinct serotypes [7]. Despite the large variety of serotypes, only small fractions have been associated with increased invasive potential and mortality [4]. As vaccine responses are highly serotype-dependent, pneumococcal vaccines were designed such that they consist of purified capsular polysaccharides from serotypes associated with IPD [5].

In Singapore, the 7-valent pneumococcal polysaccharide conjugate vaccine (PCV7) has been available on demand since 2005, and was added to the National Childhood Immunization Program (NCIP) in October 2009, but later replaced by the PCV13 in December 2011 [8]. Currently, the 10-valent Paediatric PHiD-CV, PCV13, and the 23-valent pneumococcal polysaccharide vaccine (PPV23) are the three pneumococcal vaccines available in Singapore [9,10]. The recommended pneumococcal vaccine immunization strategy is to administer PCV13 or PHiD-CV to all infants at three and five months of age, with a booster at 12 months. For adults, the recommendation is to use a single dose of PPV23 for elderly and people at higher risk of acquiring pneumococcal disease and add PCV13 at a later stage to enhance the vaccine protection [10]. Although PCV and PPV23 vaccines are recommended by the NCIP and the National Adult Immunization Program (NAIS), neither are provided free and families pay either out of pocket, through insurance or through their medical savings accounts. As it is officially recommended, providers are more likely to encourage families to pay for the vaccination, the uptake is likely to be much higher if the vaccines were provided for free [11].

However, despite inclusion of the PCVs in the NCIP in Singapore, the incidence of IPD in all age groups remains high. Although, decrease in IPD rates in children was reported a year after PCV7 implementation [11], it is yet not clear whether serotype distribution and pneumococcal disease severity and outcome changed over time in Singapore and what has been the impact on non-vaccine pneumococcal serotypes. This information will be important in the evaluation of current vaccine strategies and will provide the basis for future vaccination policies.

Here, we present a review encompassing changes in risk factors and outcome for pneumococcal infections in Singaporean adults and children spanning over 16 years.

## Methods

### Study design

We retrospectively collected clinical data on patients admitted to hospital with laboratory confirmed pneumococcal infection between January 1997 and December 2013. We reviewed records of 1,389 non-duplicate patients from four major public hospitals in Singapore: Tan Tock Seng Hospital, KK Women’s and Children’s Hospital, Singapore General Hospital and National University Hospital. Clinical data were collected from case notes and electronic medical records. Ethics for the study was approved by the Group Domain Specific Review Board (reference number 2013/01036).

### Definitions and exclusion criteria

IPD was defined as isolation of *S*. *pneumoniae* from a sterile site: blood, cerebrospinal fluid (CSF), pleural and peritoneal fluid, and others such as bone or joints. Non-invasive pneumococcal disease (non-IPD) was described when pneumococcus was recovered from a non-sterile clinical specimen such as sputum, tracheal/bronchial aspirates, and ear secretions.

Clinical syndromes were classified as follows: a) Bacteraemia: presence of a positive pneumococcal blood culture and no other documented site of infection; b) Bacteraemic pneumonia: lung infection confirmed by radiologic evidence of new-onset pulmonary infiltrates or consolidation, and a positive pneumococcal blood culture; c) Meningitis: isolation of pneumococcus from CSF, or positive pneumococcal-specific latex agglutination test on CSF sample, with or without concomitant bacteraemia; d) Other invasive infections: presence of a positive pneumococcal culture from a sterile site, excluding blood and CSF; e) Non-bacteraemic pneumonia: lung infection confirmed by radiological investigation and isolation of pneumococcus from sputum (the majority of the samples from children were obtained from suction or possible nebulised specimens), lung tissue or pleural cavity; f) Non-invasive infections: isolation of pneumococcus from a normally non-sterile site.

Immunosuppression was defined as presence of chronic renal failure, nephrotic syndrome, malignancy, solid organ transplant, or iatrogenic immunosuppression (>20mg prednisolone for >2 weeks). Critical illness was defined as having a Pitt bacteraemia score of >4 points based on values assessed 48 hours before or on the day of first positive *S*. *pneumoniae* culture [12]. Acute cardiac event during admission was defined as presenting new or worsening heart failure, arrhythmias or myocardial infarction. Adults were defined as 16 years old and older, and children were defined as under 16 years old. The analysis was limited to patients with laboratory confirmed pneumococcal disease.

### Information on pneumococcal isolates

Serotype data were available for 842 pneumococcal isolates linked to clinical cases. Serotype information was acquired from whole genome sequences (Jauneikaite *et al*, unpublished).

Antibiotic susceptibility to benzylpenicillin, ceftriaxone, erythromycin and tetracycline was tested for 482 pneumococcal isolates using E-test strips (bioMérieux, France). Isolates were defined as susceptible, intermediate susceptibility, or non-susceptible according to the interpretive standards of the Clinical and Laboratory Standards Institute [13]. In this study, isolates with intermediate susceptibility and full resistance to the named antibiotics were all grouped as non-susceptible isolates. Multi-drug resistance was reported if pneumococcal isolate was non-susceptible to at least one agent in three or more antimicrobial categories. Concordant antibiotic therapy was reported if treatment with one or more antibiotics to which a pneumococcal isolate was susceptible was given to patient within the first 48 hours of pneumococcal isolation. To further study temporal changes in serotypes and antibiotic resistance, the study was divided in three periods: period 1, years 1998–2004, pre-introduction of PCV7; and period 2, after PCV introduction. Period 2 was further divided in early period (2005–2009), where PCV7 was introduced in Singapore but mainly used in the private sector; and late period (2010–2013), after Singapore inclusion of PCV7 and later PCV13 in the NCIP.

### Statistical analysis

Statistical analyses were done using STATA v.11 (Stata Corporation, USA). Categorical findings were summarized in frequency tables. Continuous variables were expressed as means and standard deviations (SD) or as medians and interquartile ranges (IQR). Chi-square test (two-tailed) or Fisher’s exact test was used to determine the association analysis for categorical data. For continuous data, ANOVA test was used to determine statistical differences in median and t-test to determine differences in means among groups. The association between each population and covariates was assessed by univariate and multivariate logistic regression models and was expressed as an odds ratio with a 95% confidence interval (95% CI). Model covariates included those factors that were found to be significant by univariate analysis and those that had previously been found in the literature to affect the disease severity or mortality [14,15], and were selected based on a model fit aided by Akaike information criterion values [16]. A two-tailed p-value <0.05 was considered statistically significant.

## Results

### Patient population characteristics

Eight hundred eighty-nine (64%) of 1,389 cases were included in the analysis (S1 Fig). Five hundred sixty-one (63.1%) patients were adults and 328 (36.9%) were children (Table 1). Median (IQR) age was 62 (49–72) years in adults and 3 (1–5) years in children. Among them, 241 (27.1%) were aged ≥65 years and 240 (27%) <5 years. Fifty-four (16.5%) children and 22 (3.9%) adults in our cohort had received at least one dose of the appropriate pneumococcal vaccination at the time; of these 57.9% were under 5 years old (n = 44) and 10.5% of the adults were ≥65 years (n = 8). Of the vaccinated adults, 9.1% received PCV7 (n = 2), 68.2% received PPV23 (n = 15) and for 22.7% the information on vaccine type was not available (n = 5). In the children group, 68.5% received PCV7 (n = 37), 24.1% received PCV13 (n = 13), 5.5% received PPV23 (n = 3) and 1.9% we were unable to obtain the type of vaccine (n = 1).

**Table 1:** Baseline characteristics of patients with pneumococcal disease by age group.

	Total (N = 889)	Infants <12mo (n = 38)	1-2yrs (n = 95)	3–4 yrs (n = 107)	5-15yrs (n = 88)	16-64yrs (n = 320)	≥65yrs (n = 241)
**Age-median (IQR), years**	45 (4–66)	0 (0–0)	2 (1–2)	4 (3–4)	6 (5–8.5)	51 (37–59)	74 (69–80)
**Gender, n (%)**							
Male	593 (66.7)	23 (60.5)	54 (56.8)	67 (62.6)	54 (61.4)	225 (70.3)	170 (70.5)
Female	296 (33.3)	15 (39.5)	41 (43.2)	40 (37.4)	34 (38.6)	95 (29.7)	71 (29.5)
**Comorbidities, n (%)**							
At least 1 significant comorbidity	437 (49.2)	3 (7.9)	14 (14.7)	23 (21.5)	21 (23.9)	196 (61.3)	180 (74.7)
Diabetes Mellitus	120 (13.5)	0 (0.0)	0 (0.0)	0 (0.0)	0 (0.0)	56 (17.5)	64 (26.6)
Chronic Heart Disease	107 (12.0)	2 (5.3)	1 (1.1)	1 (0.9)	0 (0.0)	31 (9.7)	72 (29.9)
Immunocompromised	114 (12.8)	0 (0.0)	1 (1.05)	9 (8.4)	8 (9.1)	43 (13.4)	53 (22.0)
Smoking	95 (10.7)	0 (0.0)	0 (0.0)	0 (0.0)	0 (0.0)	61 (19.1)	34 (14.1)
Asthma	73 (8.2)	1 (2.6)	9 (9.5)	10 (9.4)	9 (10.2)	28 (8.8)	16 (6.6)
COPD	37 (4.2)	1 (2.6)	0 (0.0)	0 (0.0)	0 (0.0)	4 (1.3)	32 (13.3)
Alcoholism	33 (3.7)	0 (0.0)	0 (0.0)	0 (0.0)	0 (0.0)	25 (7.8)	8 (3.3)
Renal Insufficiency	30 (3.4)	0 (0.0)	0 (0.0)	0 (0.0)	0 (0.0)	11 (3.4)	19 (7.9)
Chronic Liver Disease	23 (2.6)	0 (0.0)	0 (0.0)	0 (0.0)	1 (1.1)	11 (3.4)	11 (4.6)
HIV	22 (2.5)	0 (0.0)	0 (0.0)	0 (0.0)	1 (1.1)	19 (5.9)	2 (0.8)
Dementia	14 (1.6)	0 (0.0)	0 (0.0)	0 (0.0)	0 (0.0)	0 (0.0)	14 (5.8)
**Living condition**							
At home	754 (84.8)	37 (97.4)	95 (100)	107 (100)	88 (100)	241 (75.3)	186 (77.2)
In nursing home	15 (1.7)	0 (0.0)	0 (0.0)	0 (0.0)	0 (0.0)	4 (1.3)	11 (4.6)
Others	20 (2.2)	1 (2.6)	0 (0.0)	0 (0.0)	0 (0.0)	17 (5.3)	2 (0.8)
Unknown	100 (11.3)	0 (0.0)	0 (0.0)	0 (0.0)	0 (0.0)	58 (18.1)	42 (17.4)
**Previous *S*. *pneumoniae* vaccination**							
No	634 (71.3)	5 (13.2)	19 (20.0)	20 (18.7)	10 (11.3)	14 (4.4)	8 (3.3)
Yes	76 (8.6)	33 (86.8)	76 (80.0)	85 (79.4)	76 (86.4)	213 (66.6)	151 (62.7)
Unknown	179 (20.1)	0 (0.0)	0 (0.0)	2 (1.9)	2 (2.3)	93 (29.0)	82 (34.0)
**Radiological**^a^							
Bilateral Infiltrates	160 (20.4)	4 (16)	9 (11.3)	16 (17.4)	13 (17.6)	58 (20)	60 (27)
Pleural effusion	249 (31.8)	2 (8.0)	23 (28.8)	33 (35.9)	28 (37.8)	86 (29.7)	77 (34.7)

Data are presented as No. (%) unless otherwise specified.

Abbreviations: IQR, interquartile range; COPD, chronic obstructive pulmonary disease; HIV, human immunodeficiency virus.

^a^Closest chest X-ray (CXR) to the positive *S*. *pneumoniae* culture, available for 25 infants, 80 children from 1-2yrs, 92 from 3-4yrs, 74 from 5-15yrs, and 290 adults from 16-64yrs and 222 ≥65yrs.

The overall ethnic distribution was: Chinese 561 (63.1%), Malays 159 (17.9%), Indian 122 (13.7%), others 47 (5.3%) and broadly reflected the national ethnic distribution (Singapore Department of Statistics, 2016).

### Clinical syndromes and outcome

**Fig 1:**
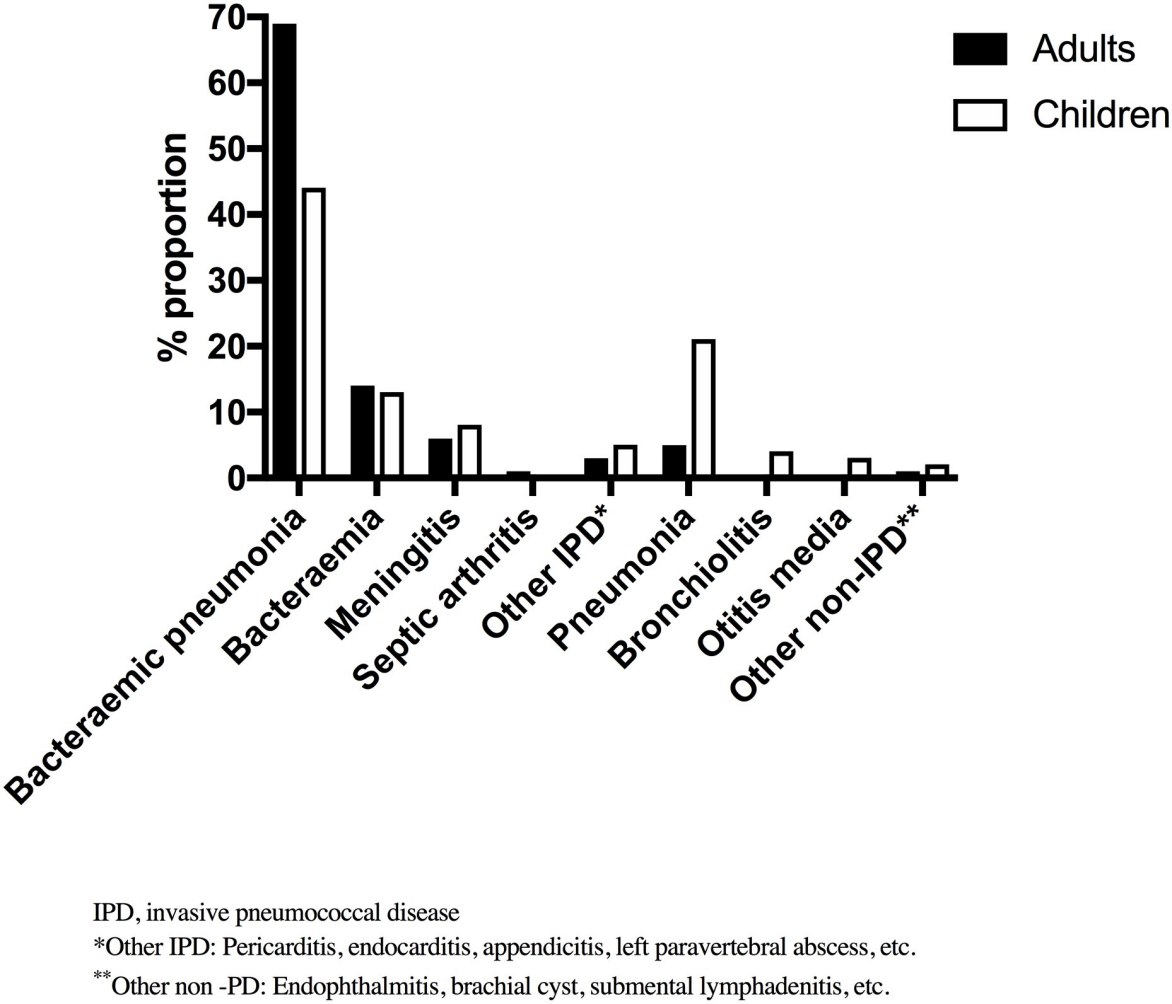
Distribution of pneumococcal disease syndromes by age group.

Bacteraemic pneumonia was the most common syndrome in adults (69.3%) and children (44.2%), followed by bacteraemia (14.3% vs. 13.4%), meningitis (6.4% vs. 7.6%) and non-bacteraemic pneumonia (5.2% vs. 21.0%) (Fig 1). Viral co-infection was more common in children group, especially in infants when compared to other children groups (Table 2). However, this did not result in infants requiring more invasive interventions compared to other older children or adult groups (Table 2). Seizures also were more commonly observed in infant group than any other group (Table 2). There were no significant differences observed between the two adult age groups when interventions or outcomes were compared. Overall, mortality rates during hospitalization were significantly higher in adults when compared to children (18.5% vs. 3.1%; P = <0.001) (Table 2). Among the adults who died, 62 (59.6%) were ≥65 years old (Table 3) and 50% of the adults below 65 years who died, had comorbidities (S1 Table). The case fatality rate was highest for pneumonia in adults (24.2%) and for meningitis in children (20%), and for infant group there were no deaths reported where data was available (Table 4).

**Table 2:** Disease outcome during hospitalization and at discharge by age group.

	Total (N = 889)	Infants <12mo (n = 38)	1-2yrs (n = 95)	3–4 yrs (n = 107)	5-15yrs (n = 88)	16-64yrs (n = 320)	≥65yrs (n = 241)	P-value
**Length of stay in hospital, days, median (IQR)**	8 (4–15)	6 (4–17)	6 (4–12)	8.5 (6–15)	10 (5–16)	7.5 (4–15)	8 (4–15)	0.360^1^
**Viral Co-infection**	44 (5.0)	9 (23.7)	11 (11.7)	8 (7.5)	6 (6.8)	10 (3.1)	0 (0.0)	<0.001^2^
**Required ICU, n (%)**	194 (21.8)	6 (15.8)	16 (16.8)	26 (24.3)	23 (26.1)	79 (24.7)	44 (18.3)	0.923^2^
**Length of stay in ICU, days, median (IQR)**	3 (2–8)	3.5 (2–6)	2 (1–4)	2 (1–8)	5 (3–13)	3 (2–6)	3 (1.5–7)	0.151^1^
**Critical illness (PBS>4)**	72 (8.1)	2 (5.3)	5 (5.3)	8 (7.5)	6 (6.8)	30 (9.4)	21 (8.7)	0.775^2^
**Invasive interventions**								
Supplemental oxygen	383 (43.1)	8 (21.6)	29 (30.5)	41 (38.3)	37 (42.0)	128 (40.0)	140 (58.1)	<0.001^2^
Non-invasive mechanical ventilation	79 (8.9)	2 (5.4)	11 (11.6)	17 (15.9)	16 (18.2)	13 (4.1)	20 (8.3)	<0.001^2^
Invasive mechanical ventilation	153 (17.2)	5 (13.5)	14 (14.8)	13 (12.2)	14 (15.9)	67 (21.0)	40 (16.6)	0.309^2^
Renal replacement therapy	26 (2.9)	0 (0.0)	1 (1.1)	2 (1.9)	3 (3.4)	12 (3.8)	8 (3.3)	0.597^2^
Inotropes/vasopressors	102 (11.5)	1 (2.7)	2 (2.1)	9 (8.4)	10 (11.4)	42 (13.1)	38 (15.8)	0.004^2^
Blood transfusion or products	68 (7.7)	0 (0.0)	8 (8.4)	13 (12.2)	13 (14.8)	19 (6.0)	15 (6.2)	0.013^3^
Surgical procedures	238 (26.8)	6 (16.2)	29 (30.5)	42 (39.3)	41 (46.6)	71 (22.2)	49 (20.3)	<0.001^2^
**Clinical outcome**								
Non-pneumococcal nosocomial complication	86 (9.7)	2 (5.3)	9 (9.5)	13 (12.2)	12 (13.6)	28 (8.8)	22 (9.1)	0.509^2^
ARDS	25 (2.8)	0 (0.0)	0 (0.0)	3 (2.8)	5 (5.7)	10 (3.1)	7 (2.9)	0.256^3^
Pneumothorax	23 (2.6)	1 (2.6)	2 (2.1)	6 (5.6)	5 (5.7)	5 (1.6)	4 (1.7)	0.080^3^
Seizures	35 (3.9)	5 (13.2)	6 (6.3)	8 (7.5)	0 (0.0)	11 (3.4)	5 (2.1)	0.002^3^
Stroke	7 (0.8)	0 (0.0)	0 (0.0)	1 (0.9)	0 (0.0)	2 (0.6)	4 (1.7)	0.721^3^
Acute cardiac events	84 (9.5)	1 (2.6)	1 (1.1)	2 (1.9)	2 (2.3)	31 (9.70	47 (19.5)	<0.001^3^
Coagulopathy or DIC	52 (5.9)	0 (0.0)	4 (4.2)	5 (4.7)	5 (5.7)	22 (6.9)	16 (6.6)	0.620^3^
Rhabdomyolisis	14 (1.6)	0 (0.0)	1 (1.1)	0 (0.0)	0 (0.0)	7 (2.2)	6 (2.5)	0.454^3^
Acute renal injury	148 (16.7)	0 (0.0)	3 (3.2)	4 (3.7)	7 (8.0)	71 (22.2)	63 (26.1)	<0.001^2^
Gastrointestinal bleeding	10 (1.1)	0 (0.0)	0 (0.0)	2 (1.9)	0 (0.0)	6 (1.9)	2 (0.8)	0.586^3^
Hepatic dysfunction	45 (5.1)	0 (0.0)	1 (1.1)	2 (1.9)	4 (4.6)	19 (5.9)	19 (7.9)	0.035^3^
Hyperglycemia	22 (2.5)	0 (0.0)	0 (0.0)	2 (1.9)	1 (1.1)	12 (3.8)	7 (2.9)	0.355^3^
Hypoglycemia	14 (1.6)	0 (0.0)	1 (1.1)	1 (0.9)	0 (0.0)	7 (2.2)	5 (2.1)	0.825^3^
**Discharge outcome**								
Alive without sequelae	676 (76.1)	35 (92.1)	87 (91.6)	95 (88.8)	78 (88.6)	235 (73.5)	146 (60.6)	
Alive with sequelae	99 (11.1)	3 (7.9)	5 (5.2)	8 (7.5)	7 (8.0)	43 (13.4)	33 (13.7)	
Dead	114 (12.8)	0 (0.0)	3 (3.2)	4 (3.7)	3 (3.4)	42 (13.1)	62 (25.7)	<0.001^2^
**Concordant therapy**^a^	247 (83.2)	5 (83.3)	11 (36.7)	17 (54.8)	16 (64.0)	116 (99.2)	82 (93.2)	<0.001^2^
**Time to antibiotics, days, median (IQR)**^b^	0 (0–0)	0 (0–1)	0 (0–1)	0 (0–0)	0 (0–1)	0 (0–0)	0 (0–0)	<0.665^1^
**Time to death from admission, days, median (IQR)**	3 (1–10)	NA	1 (0–4)	0 (0–0.5)	16 (5–42)	3 (1–9)	3 (1–10)	0.265^1^

Data are presented as No. (%) unless otherwise specified.

Abbreviations: IQR, interquartile range; ICU, intensive care unit; PBS, Pitt bacteraemia score; ARDS, acute respiratory distress syndrome; DIC, disseminated intravascular coagulation; NA, non-applicable.

^1^ ANOVA test;

^2^Chi-squared test;

^3^Fisher’s exact test.

^a^ Prescribed medication and MIC available for 205 adults and 92 children.

^b^ Prescribed medication available for 485 adults and 285 children.

**Table 3:** Factors associated with disease outcome at discharge in Adults with *S*. *pneumoniae* infection.

	Cured (%)	Discharged w/ sequelae (%)	Death (%)	OR, univariate (95% CI)	P-value	OR, multivariate (95% CI)	P-value
	n = 381	n = 76	n = 104				
Gender*							
Male	260 (65.8)	56 (14.2)	79 (20.0)	1			
Female	121 (72.9)	20 (12.0)	25 (15.1)	0.7 (0.4–1.2)	0.171		
Age group, years*							
16–64	235 (73.4)	43 (13.4)	42 (13.1)	1			
≥65	146 (60.6)	33 (13.7)	62 (25.7)	2.3 (1.5–3.5)	<0.001	2.5 (1.4–4.6)	0.002
**Type of PD**							
Bacteremic pneumonia	264 (67.9)	47 (12.1)	78 (20.0)	1			
Bacteremia	61 (76.3)	6 (7.5)	13 (16.2)	0.8 (0.4–1.5)	0.435		
Meningitis	23 (63.9)	7 (19.4)	6 (16.7)	0.8 (0.3–2.0)	0.626		
Others IPD	15 (71.4)	6 (28.6)	0 (0.0)				
Pneumonia	13 (44.8)	9 (31.0)	7 (24.2)	1.3 (0.5–3.1)	0.599		
Others non IPD	5 (83.3)	1 (16.7)	0 (0.0)				
**Comorbidities**							
Any*	259 (68.9)	52 (13.8)	65 (17.3)	0.8 (0.5–1.2)	0.278		
CHD	74 (71.9)	13 (12.6)	16 (15.5)	0.7 (0.4–1.4)	0.386		
Asthma	35 (79.5)	5 (11.4)	4 (9.1)	0.4 (0.1–1.2)	0.103		
COPD*	29 (80.5)	5 (13.9)	2 (5.6)	0.2 (0.1–1.0)	0.055	0.1 (0.0–0.8)	0.023
Renal Insufficiency	19 (63.3)	3 (10.0)	8 (26.7)	1.6 (0.7–3.8)	0.243		
Chronic liver disease	11 (50.0)	5 (22.7)	6 (27.3)	1.7 (0.6–4.4)	0.287		
Immunocompromised	63 (65.6)	13 (13.6)	20 (20.8)	1.2 (0.7–2.1)	0.525		
HIV	17 (80.9)	3 (14.3)	1 (4.8)	0.2 (0.1–1.6)	0.132		
Diabetes mellitus	85 (70.8)	14 (11.7)	21 (17.5)	0.9 (0.5–1.6)	0.741		
Dementia*	5 (35.7)	0 (0.0)	9 (64.3)	8.5 (2.8–26.1)	<0.001	4.2 (1.2–14.8)	0.024
Alcohol consumption	19 (57.6)	6 (18.2)	8 (24.2)	1.4 (0.6–3.2)	0.39		
Smoking	65 (68.4)	17 (17.9)	13 (13.7)	0.7 (0.3–1.2)	0.181		
**Clinical**							
Fever*	295 (75.1)	41 (10.4)	57 (14.5)	0.4 (0.3–0.7)	<0.001	0.5 (0.3–0.8)	0.009
Chest pain*	115 (79.9)	20 (13.9)	9 (6.2)	0.2 (0.1–0.5)	<0.001	0.3 (0.1–0.7)	0.006
Acute cardiac events*	38 (48.7)	14 (18.0)	26 (33.3)	2.6 (1.5–4.4)	<0.001	1.9 (0.9–3.9)	0.064
Critical illness (PBS>4)*	8 (15.7)	6 (11.8)	37 (72.5)	17.5 (9.0–34.0)	<0.001	17.8 (8.0–39.6)	<0.001
Bilateral Infiltrates^a^*	63 (53.4)	13 (11.0)	42 (35.6)	3.4 (2.1–5.5)	<0.001	2.9 (1.6–5.1)	<0.001
Pleural effusion^a^*	135 (59.7)	44 (19.5)	47 (20.8)	1.7 (1.1–2.6)	0.028		
**Treatment**							
Discordant therapy	5 (71.4)	2 (28.6)	0 (0.0)				
Penicillin resistance^b^	66 (60)	17 (15.5)	27 (24.5)	1.6 (0.9–2.8)	0.107		
MDR^b^	11 (55.0)	5 (25.0)	4 (20.0)	1.1 (0.3–3.4)	0.89		

Data are presented as No. (%) unless otherwise specified.

Abbreviations: PD, pneumococcal disease; IPD, invasive pneumococcal disease; CHD, chronic heart disease; COPD, chronic obstructive pulmonary disease; HIV, human immunodeficiency virus; MDR, multidrug resistance.

^a^ Chest X-ray available for 512 adults during admission.

^b^ MIC available for 324 adults.

*Variables tested on multivariate analysis.

**Table 4:** Factors associated with disease outcome at discharge in children with *S*. *pneumoniae* infection.

	Cured (%)	Discharged w/ sequelae (%) n = 22	Death (%)	OR, univariate (95% CI)	P-value	OR, multivariate (95% CI)	P-value
	n = 295		n = 10				
Gender*							
Male	185 (93.4)	10 (5.1)	3 (1.5)	1			
Female	110 (84.6)	13 (10)	7 (5.4)	3.7 (0.9–14.5)	0.063		
Age group*							
Infants <12mo	35 (92.1)	3 (7.9)	0 (0.0)				
1-2yrs	87 (91.6)	5 (5.3)	3 (3.2)	0.8 (0.2–3.9)	0.822		
3-4yrs	95 (88.8)	8 (7.5)	4 (3.7)	1			
5-15yrs	78 (88.6)	7 (8.0)	3 (3.4)	0.9 (0.2–4.2)	0.902		
**Type of PD**							
Bacteremic pneumonia	134 (92.4)	7 (4.8)	4 (2.8)	1			
Bacteremia	43 (97.7)	1 (2.3)	0 (0)				
Meningitis*	13 (52)	7 (28)	5 (20)	8.8 (2.2–35.6)	0.002	17.2 (1.6–187.5)	0.019
Others IPD	16 (94.1)	1 (5.9)	0 (0)				
Pneumonia	63 (91.3)	5 (7.3)	1 (2.5)	0.5 (0.1–4.7)	0.56		
Others non IPD	26 (92.9)	2 (7.1)	0 (0)				
**Comorbidities**							
Any*	50 (81.9)	7 (11.5)	4 (6.6)	3.1 (0.8–11.2)	0.092	6.1 (0.8–46.6)	0.083
CHD	3 (75)	1 (25)	0 (0)				
Asthma	26 (89.7)	3 (10.3)	0 (0)				
Immunocompromised	18 (100)	0 (0)	0 (0)				
HIV	0 (0)	0 (0)	1 (100)				
Chronic liver disease	0 (0)	0 (0)	1 (100)				
**Clinical**							
Fever	272 (91.0)	19 (6.3)	9 (3.0)	0.8 (0.1–6.8)	0.867		
Chest pain	20 (100)	0 (0)	0 (0)				
Acute cardiac events*	2 (33.3)	1 (16.7)	3 (50)	45.0 (7.7–263.4)	<0.001		
Critical illness (PBS>4)*	7 (33.3)	7 (33.3)	7 (33.3)	50.7 (11.8–217.0)	<0.001	24.0 (3.4–171.5)	0.002
Bilateral Infiltrates^a^*	32 (76.2)	4 (9.5)	6 (14.3)	9.4 (2.5–34.9)	0.001	11.9 (1.2–117.5)	0.034
Pleural effusion^a^	93 (87.7)	9 (8.5)	4 (3.8)	0.5(0.1–2.5)	0.424		
**Treatment**							
Discordant therapy	43 (100)	0 (0)	0 (0)				
Penicillin resistance^b^	100 (91.8)	7 (6.4)	2 (1.8)	0.5 (0.0–6.0)	0.602		
MDR^b^	46 (86.8)	6 (11.3)	1 (1.9)	0.9 (0.1–10.1)	0.928		

Data are presented as No. (%) unless otherwise specified.

Abbreviations: PD, pneumococcal disease; IPD, invasive pneumococcal disease; CHD, chronic heart disease; COPD, chronic obstructive pulmonary disease; HIV, human immunodeficiency virus; MDR, multidrug resistance.

^a^ Chest X-ray available for 271 children during admission.

^b^ MIC available for 148 children.

*Variables tested on multivariate analysis.

### Risk factors for pneumococcal disease mortality

In adult patients, multivariate logistic regression showed that age ≥65 years (OR 2.5, 95% CI 1.4–4.6; P = 0.002), dementia (OR 6.8, 95% CI 2.0–23.3; P = 0.002), acute cardiac events (OR 2.4, 95% CI 1.2–4.7; P = 0.013), critical illness (OR 14.2, 95% CI 6.7–30.2; P = <0.001) and multilobar pulmonary involvement (OR 3.0, 95% CI 1.7–5.3; P = <0.001) were significantly associated with death (Table 3). In children, multivariate analysis revealed that the risk for death was higher for patients with meningitis (OR 17.2, 95% CI 1.6–187.5; P = 0.019), critical illness (OR 24.0, 95% CI 3.4–171.5; P = 0.002), and bilateral infiltrates (OR 11.9, 95% CI 11.2–117.5; P = 0.034) (Table 4). Inferences were not altered in the sensitivity analysis where only patients with IPD were included to address bias (S2 and S3 Tables).

#### Pneumococcal serotype and antimicrobial susceptibility changes in relation to disease syndrome and time period

**Fig 2:**
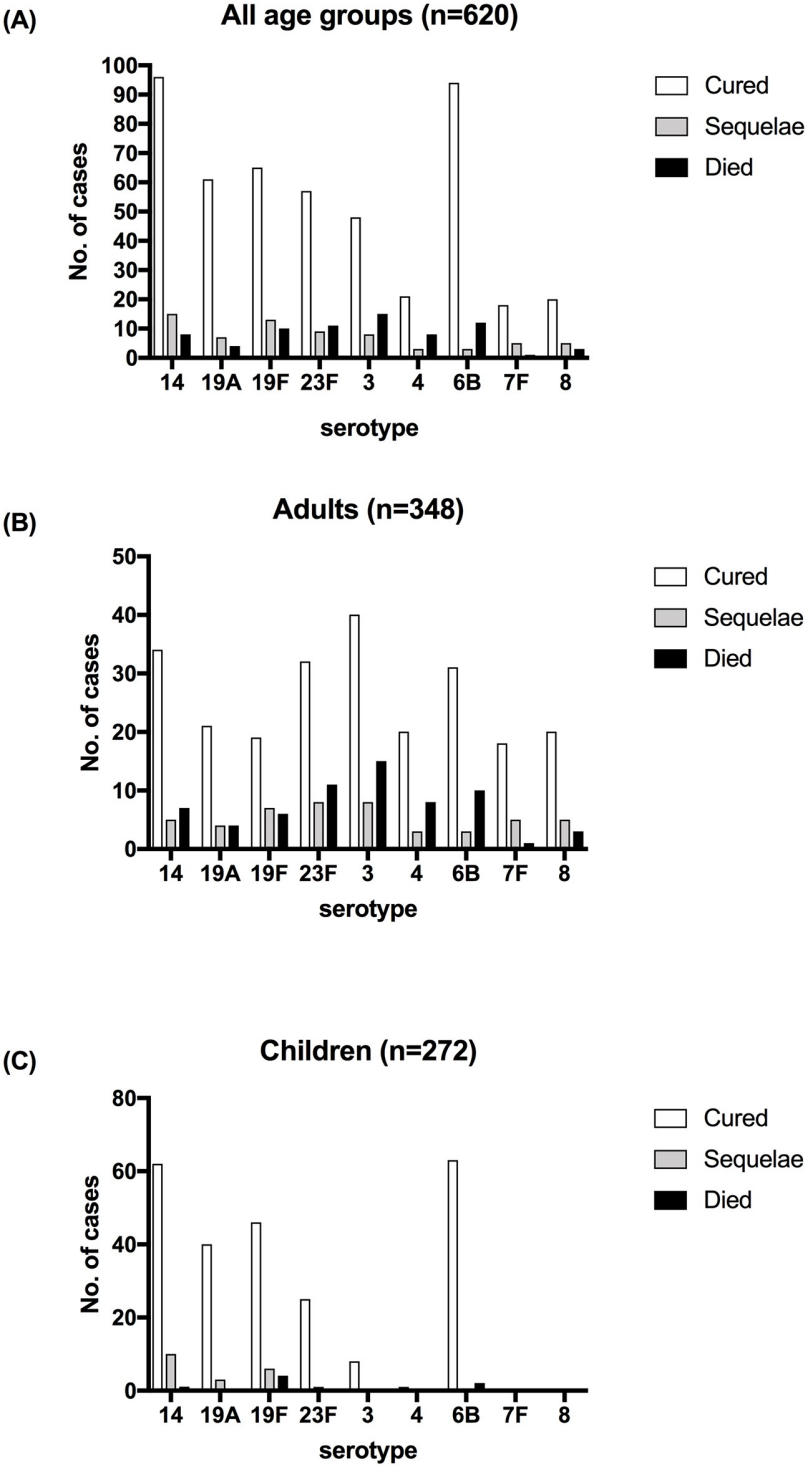
Disease outcome at hospital discharge by age and serotype for patients with pneumococcal disease. Number of reported cases where the outcome and the pneumococcal serotype were known: (A) overall cases in the study; (B) adult only cases (>16 years old) and (C) children only cases (<16 years old).

Serotypes 3, 23F, 14, 6B, 19F and 4 were most commonly identified in adult pneumococcal disease, and 14, 6B and 19F were most common in children (Fig 2). Serotype 6B was most prevalent in adults with bacteraemia without focus (17%) and one of the most prevalent in meningitis along with serotypes 1 and 23F (11%). Serotype 14 was the most prevalent in children with meningitis, bacteraemia and bacteraemic pneumonia followed by serotype 6B (S4 and S5 Tables). Serotypes 3, 23F, 6B, 14 and 19F were identified in ~50% of the adult fatalities, with serotype 3 identified in 24% alone (15 out of 63 cases). In children, serotypes 19F and 6B were identified in 60% of the deaths, with the highest case fatality rate for serotype 19F (4 deaths for 56 cases).

We further compared the changes in composition of serotypes between the three study periods. There was a significant difference in serotype composition between period 1 and both the early and late PCV introduction periods in both groups (S6 and S7 Tables). Serotype 19A increased by more than 5-fold in both groups from period 1 to period 2 (1.6% to 5.6% in adults and from 4.5% to 20.8% in children).

Data on antibiotic susceptibility were available for 472 pneumococcal isolates (Table 5). The prevalence of antimicrobial resistance was higher in pneumococcal isolates from children than adults. Resistance to penicillin was reported in 79% of children and 35.4% of adult cases (P = <0.001). Pneumococcal isolates from children were more likely to be non-susceptible to multiple antibiotics when compared to isolates from adults (51.4% vs. 10.2%, respectively; P = <0.001). There was not significant difference in antibiotic susceptibility between the time periods probably due to the small numbers of antibiotic susceptibility tests available for isolates from the first period.

**Table 5:** Antibiotic susceptibility results for S. pneumoniae by age group.

	Adult ≥ 16yrs			Children <16yrs			P-value^1^
	Susceptible	Non-susceptible	Non-susceptible serotypes n(%)^2^	Susceptible	Non-susceptible	Non-susceptible serotypes n(%)^2^	
Penicillin	201/311 (64.6)	110/311 (35.4)	1: 1(17); **14: 18(72)**; **15A: 4 (80)**; **19A: 18(56)**; **19F: 16(84)**; **23A: 6(67)**; **23F: 21(68)**; 3: 4(13); **35B: 1(100)**; 38: 1(50); 4: 1(5); 6A: 2(50); 6B: 12(50); 6C: 1(11); 8: 2(9); **9A: 2(100)**	29/138 (21.0)	109/138 (79.0)	**11D: 1(100)**; **14: 19(59)**; **15A: 1(100)**; **19A: 23(92)**; **19F: 27(96)**; **20: 1(100)**; **23A: 1(100)**; **23F: 14(82)**; 3: 1(40); **4: 1(100)**; 6A: 3(37.5); **6B: 14 (88)**	<0.001
Ceftriaxone	261/269 (97.0)	8/269 (3.0)	19A: 1(6.3); 19F: 3(17); 23F: 2(7); 6A: 1(25); 8: 1(6.3)	126/137 (92.0)	11/137 (8.0)	14: 1(4); 19A: 4(14); 19F: 3(10); 6B: 3(16)	0.023
Tetracycline	35/78 (44.9)	43/78 (55.1)	**12F: 2(100)**; 14: 2(40); **15A: 1(100)**; **18B/C: 1(100)**; **18F: 1(100)**; 19A: 2(40); **19F: 5(100)**; 20: 1(50); 23A: 1(25); **23F: 11(85)**; 3: 4(40); **39: 1(100)**; **4: 3(100)**; **6A: 1(100)**; 6B: 3(43); **6C: 1(100)**; 7F: 1(50); 8: 2(33)	39/104 (37.5)	65/104 (62.5)	14: 9(45); **15A: 2(100)**; **19A: 17(65)**; **19F: 21(84)**; **20: 1(100)**; **23F: 9(69)**; **6B: 6(55)**	0.316
Erythromycin	38/64 (59.4)	26/64 (40.6)	**14: 2(100)**; **15A: 1(100)**; **18F: 1(100)**; 19A: 1(50); **19F: 3(75)**; 23A: 1(25); **23F: 9(75)**; 3: 2(22); 4: 1(33); **6A: 1(100)**; 6B: 2(40); **6C: 1(100)**; 8: 1(20)	15/89 (16.9)	74/89 (83.2)	**14: 15(83)**; **15A: 1(100)**; **19A: 20(91)**; **19F: 22(92)**; **20: 1(100)**; **23F: 9(75)**; 3: 1(50); 6A: 1(25); **6B: 4(80)**	<0.001

Data are presented as No. (%) unless otherwise specified.

^1^Chi-squared test

^2^Proportion of resistant isolates per total isolates of a given serotype. Serotypes with more than 50% of isolates resistant are shown in bold.

Among the 110 adult isolates non-susceptible to penicillin, 21 (19%) were of serotype 23F. This serotype also accounted for the higher number of tetracycline resistance (26%). Serotype 19F was frequently non-susceptible to penicillin (16 isolates (84%)), tetracycline (5 isolates (100%)) and erythromycin (3 isolates (75%)).

Overall, infections caused by the serotypes included in PCV7 accounted for 37% of the adult IPD cases and 67% of children’ IPD cases. Pneumococcal disease caused by the additional six serotypes included in PCV13 represented a further 25% of the adults and 21% of children’s cases. Also, 8% of adults and 1% of children pneumococcal disease cases were attributed to the additional serotypes included in PPV23, while 30% and 11% corresponded to serotypes currently not included in any of the pneumococcal vaccines in adults and children, respectively.

## Discussion

This is the first large-scale retrospective study investigating differences in pneumococcal disease and clinical outcomes between adult and paediatric populations in Singapore. Data collected between 1997 and 2013 showed that mortality due to pneumococcal disease was high, with up to 19% case fatality in adults and 3% in children. Bacteraemic pneumonia was the most frequent clinical pneumococcal disease syndrome present in hospitalized patients. Overall 63% of pneumococcal isolates from adults and 88% isolates from children were serotypes included in PCV13, however, the vaccination rates in studied populations were very low. Approximately, 30% of serotypes identified in the study as causing disease were not covered in any licensed vaccine.

We found that presenting syndromes such as critical illness, bilateral infiltrates on chest imaging or dementia in adults and meningitis in children, were independent risk factors significantly associated with mortality. Critical illness was the most important risk factor for death in both groups, which is in agreement with other reported studies [15]. We did not find immunosuppression, especially HIV infection, significantly associated with death as previously shown in other literature [15], most probably due to the low HIV infection prevalence in this cohort, although some cases of primary bacteraemia due to the pneumococcus may have been in undiagnosed HIV positive individuals. Elderly patients presented a higher risk for death than the younger adults, but no differences were found among children groups. Interestingly, the mortality rates of severe pneumococcal cases were independent of the concordance of therapy initiated or the pneumococcal isolate’s antibiotic susceptibility.

The introduction of conjugated pneumococcal vaccines in children has led to a significant decline in incidence of IPD in all age groups, most likely due to herd effect from immunizing infants, reducing nasopharyngeal colonization and transmission to other children and adults in other countries [17,18] but this has not been the case in Singapore to our knowledge as the majority of the cases described in this study were not vaccinated. Even though PCV7 for children was introduced to the national immunization program in Singapore in 2009 and has also been recommended for adults with comorbidities and the elderly (>65 years and older), only a small proportion of them were vaccinated. Since the introduction of pneumococcal vaccines to the NCIP, the vaccination uptake has been slowly increasing from 21.6% in 2009 to 60% in 2012 [19] reaching coverage of two doses of PCV in Singapore by the age 1 year of 79.6%, and coverage of the booster dose by age 2 years of 58.9% in 2013 [11]. However, the costs of vaccination are still to be covered by the patient which could in partly explain the low levels of vaccination. This and high adult mortality rate in our study reflect an urgent need to address this issue and rethink the current pneumococcal vaccination strategies in the country. In our study, serotype 19A increased dramatically in both children and adults in the post-PCV7 period. This is a great concern because we also found a high proportion of penicillin-non-susceptible strains among this serotype. Serotype replacement in IPD was observed after widespread use of PCV7 and this replacement has been dominated by penicillin non-susceptible serotype, 19A in several countries [20,21], hence, it is not surprising that the rise has been seen in Singapore too, especially when vaccination uptakes rates were fairly low. Although, this serotype, is already included in PCV13, it should be closely monitored in the future as well as any other non-vaccine serotypes.

Antibiotic resistance in *S*. *pneumoniae* has increased greatly worldwide over the last decade [22–24]. In Singapore, the resistance rate of 62% for paediatric isolates was reported in 2004, while 47.5% of adult isolates were non-susceptible in 2001 [22,25]. Here, we showed higher resistance rates in disease-causing pneumococcal isolates with 79% of paediatric pneumococcal isolates and 35.4% of adult isolates being non-susceptible to penicillin. This increase seems to be serotype dependent, likely due to the emergence of widely drug non-susceptible serotype 19A after the PCV7 introduction in Singapore. Likewise, erythromycin resistance has increased substantially from previous reports, reaching 40.6% in adults and 83.2% in children, probably correlated with an increase in macrolide consumption [26]. Children have been reported as the major reservoir of *S*. *pneumoniae* and they are also more susceptible to pneumococcal disease caused by non-susceptible strains [27,28], potentially explaining why children from our study were also more likely to be infected with pneumococcal strains non-susceptible to at least one antibiotic. This could also be a result of the likelihood that children in Singapore were more likely to be prescribed antibiotics than adults in our study.

However, despite this dramatic escalation, analyzing the impact of antimicrobial resistance *per se* on clinical outcomes is difficult. MDR and penicillin resistance were not associated with increased mortality among patients with pneumococcal disease in this study, where the vast majority od isolates had a penicillin MIC below 4mg/dl. Previous studies have shown that antimicrobial resistance to penicillin can be overcome with increasing doses of penicillin and thus the growing threat of antibiotic resistance has not yet become clinically relevant in the management of pneumococcal pneumonia [15].

We also found that discordant antimicrobial regimens prescribed at admission for patients with drug non-susceptible isolates was not significantly associated with a higher mortality rate. This suggests that mortality in patients with pneumococcal disease could be related to other host factors or pathogen virulence determinants rather than antibiotic sensitivity or early treatment adequacy [29].

Overall mortality rate in this study was remarkably higher in the adults (18.5% vs. 3.1%, P<0.001), similar to others [30–32]. Similar to prior smaller studies from Singapore, we found that the main cause of death in adults was pneumonia and in children was meningitis [25,33]. Surprisingly, the presence of meningitis was only significantly associated with death in the paediatric population, but not in adults. This could have been related to the small numbers of adult meningitis cases, probably underestimated in our study. The case fatality rate was higher for vaccine serotypes 4, 3 and 6B in adults and 19F and 6B in children, in agreement with previous reports from Belgium, the Netherlands and the US [34,35]. Half of the fatal cases in the adults under 65 years old took place in patients without predisposing underlying conditions, and therefore, without a recognized pneumococcal vaccine indication by current guidelines. The rates of vaccination in our cohort are so low that it is hard to determine the impact on the elderly but the data on invasive disease suggests that the potential to have a marked impact is significant as this has been seen in other developed countries [36]. Collectively, the higher mortality rates in the adult group strongly suggest the need to strengthen vaccination efforts since vaccination of at-risk adults only and elderly may not have the desired effect on mortality.

The other striking finding in our study was that 30% of isolates in adults including 18.6% of fatal cases were serotypes not covered by any of the current vaccines. This has raised the concern about the limitations of current vaccines and shows that serotype diversity can be seen even in a population with a low vaccine uptake. This also furthers the need to improve on current vaccines in terms of coverage.

Our study has several limitations. Firstly, the pneumococcal isolates were provided by the laboratories depending on their availability, therefore, collected clinical data and serotype distributions do not represent the overall prevalence of pneumococcal disease in Singapore. Secondly, we were able to carry out phenotypic antibiotic susceptibility testing only on half the available pneumococcal isolates included in the study, though isolates selected for testing were randomly selected from range of patient ages to indicate the potential distribution of the antibiotic susceptible and non-susceptible isolates. Thirdly, although the syndrome classification in our study required the microbiological results and its radiological and clinical support, a misclassification might have occurred. Lastly, changes in guidelines for blood culture may have occurred during the study period and some patients may have received antibiotic therapy before obtaining blood samples and for these patients there is a possibility that pneumococcal culture was not reported.

In conclusion, host factors and responses to the illness play an important role in mortality, and those are different in adults and children. Our results agree with global reports that the course of pneumococcal disease and its clinical outcome were more severe in elderly adults than in children. Although many of the main serotypes causing invasive disease were covered by the vaccines in use, it is imperative to continue vigilance for emergence of novel serotypes and development of vaccines with expanded coverage. The high mortality rates in this study reflect an urgent need to increase vaccination coverage in both adults and children to tackle this vaccine-preventable infection.

## Supporting information

S1 Fig Patient enrollment. (DOCX)

S1 Table Dead patients and comorbidities, by age group. (DOCX)

S2 Table Factors associated with disease outcome at discharge in adults with invasive S. pneumoniae infection. (DOCX)

S3 Table Factors associated with disease outcome at discharge in children with invasive S. pneumoniae infection. (DOCX)

S4 Table Distribution of pneumococcal serotypes and clinical presentation in adults. (DOCX)

S5 Table Distribution of pneumococcal serotypes and clinical presentation in children. (DOCX)

S6 Table Changes in proportion of adults PD serotypes from period 1 to period 2. (DOCX)

S7 Table Changes in proportion of children PD serotypes from period 1 to period 2. (DOCX)

S1 Dataset (XLSX)

S1 Codebook (DOC)
