# Supplementary material for: Risk factor profiles and clinical outcomes for children and adults with pneumococcal infections in Singapore: A need to expand vaccination policy?
Source: PLoS One. 2019 Oct 16;14(10):e0220951. doi: 10.1371/journal.pone.0220951 (PMC6795432; doi:10.1371/journal.pone.0220951)
Supplement: S1 Fig — (DOCX) [file pone.0220951.s001.docx]

**Supplementary Figure 1. Flowchart of available patient data inclusion in the study.**
