# Supplementary material for: Risk factor profiles and clinical outcomes for children and adults with pneumococcal infections in Singapore: A need to expand vaccination policy?
Source: PLoS One. 2019 Oct 16;14(10):e0220951. doi: 10.1371/journal.pone.0220951 (PMC6795432; doi:10.1371/journal.pone.0220951)
Supplement: S1 Table — (DOCX) [file pone.0220951.s002.docx]

**Supplementary Table 1. Dead patients and comorbidities, by age group**

|  | **At least 1 comorbidity (%)**  **n=258** | **No comorbidities (%)**  **n=45** | **P-value** |
| --- | --- | --- | --- |
| **Age group, years**  <5  5-15  16-64  ≥65 | 2 (28.6)  2 (66.7)  21 (50)  44 (71) | 5 (71.4)  1 (33.3)  21 (50)  18 (29) | 0.050 |
