# Supplementary material for: Risk factor profiles and clinical outcomes for children and adults with pneumococcal infections in Singapore: A need to expand vaccination policy?
Source: PLoS One. 2019 Oct 16;14(10):e0220951. doi: 10.1371/journal.pone.0220951 (PMC6795432; doi:10.1371/journal.pone.0220951)
Supplement: S2 Table — (DOCX) [file pone.0220951.s003.docx]

Supplementary Table 2. Factors associated with disease outcome at discharge in adults with invasive *S. pneumoniae* infection.

|  | **Cured (%)**  **n=366** | **Discharged w/ sequelae (%)**  **n=67** | **Death (%)**  **n=99** | **OR, univariate (95% CI)** | **P-value** | **OR, multivariate (95% CI)** | **P-value** |
| --- | --- | --- | --- | --- | --- | --- | --- |
| **Gender** |  |  |  |  |  |  |  |
| Male | 248 (66.7) | 47 (12.6) | 77 (20.7) | 1.0 |  |  |  |
| Female* | 118 (73.8) | 20 (12.5) | 22 (13.7) | 0.6 (0.4-1.0) | 0.061 |  |  |
| **Type of IPD** |  |  |  |  |  |  |  |
| Bacteremic pneumonia | 264 (67.9) | 47 (12.1) | 78 (20.0) | 1 |  |  |  |
| Bacteremia | 61 (76.3) | 6 (7.5) | 13 (16.2) | 0.8 (0.4-1.5) | 0.435 |  |  |
| Meningitis | 23 (63.9) | 7 (19.4) | 6 (16.7) | 0.8 (0.3-2.0) | 0.626 |  |  |
| Others | 15 (71.4) | 6 (28.6) | 0 (0.0) |  |  |  |  |
| Pneumonia | 3 (50) | 1 (16.7) | 2 (33.3) | 2.0 (0.4-11.0) | 0.431 |  |  |
| **Comorbidities** |  |  |  |  |  |  |  |
| Any* | 251 (69.7) | 46 (12.8) | 63 (17.5) | 0.8 (0.5-1.3) | 0.342 |  |  |
| CHD | 72 (73.5) | 11 (11.2) | 15 (15.3) | 0.8 (0.4-1.4) | 0.353 |  |  |
| Asthma* | 33 (80.5) | 5 (12.2) | 3 (7.3) | 0.3 (0.1-1.1) | 0.065 |  |  |
| COPD* | 27 (81.8) | 4 (12.1) | 2 (6.1) | 0.3 (0.1-1.1) | 0.074 | 0.2 (0.0-1.2) | 0.072 |
| Renal Insufficiency | 18 (62.1) | 3 (10.3) | 8 (27.6) | 1.7 (0.7-4.0) | 0.206 |  |  |
| Chronic liver disease | 11 (50.0) | 5 (22.7) | 6 (27.3) | 1.7 (0.6-4.4) | 0.291 |  |  |
| Immunocompromised | 62 (66.0) | 12 (12.7) | 20 (21.3) | 1.2 (0.7-2.1) | 0.464 |  |  |
| HIV | 17 (80.9) | 3 (14.3) | 1 (4.8) | 0.2 (0.0-1.6) | 0.131 |  |  |
| Diabetes mellitus | 83 (72.8) | 11 (9.7) | 20 (17.5) | 0.9 (0.5-1.6) | 0.742 |  |  |
| Dementia* | 3 (25) | 0 (0.0) | 9 (75) | 14.3 (3.8-54) | <0.001 | 13.6 (3.2-57.7) | <0.001 |
| Alcohol consumption | 18 (58.1) | 5 (16.1) | 8 (25.8) | 1.6 (0.7-3.6) | 0.292 |  |  |
| Smoking | 63 (68.5) | 16 (17.4) | 13 (14.1) | 0.7 (0.4-1.3) | 0.227 |  |  |
| **Clinical** |  |  |  |  |  |  |  |
| Fever* | 291 (75) | 40 (10.3) | 57 (14.7) | 0.4 (0.3-0.7) | <0.001 | 0.4 (0.2-0.7) | 0.001 |
| Chest pain* | 115 (79.9) | 20 (13.9) | 9 (6.2) | 0.2 (0.1-0.5) | <0.001 | 0.3 (0.1-0.6) | 0.003 |
| Acute cardiac events* | 37 (50.7) | 12 (16.4) | 24 (32.9) | 2.5 (1.5-4.3) | 0.001 | 2.5 (1.2-5.2) | 0.012 |
| Critical illness (PBS>4)* | 8 (16.7) | 4 (8.3) | 36 (75) | 20 (9.9-40.6) | <0.001 | 18.0 (8.0-40.2) | <0.001 |
| Bilateral Infiltrates^a^* | 59 (54.1) | 10 (9.2) | 40 (36.7) | 3.6 (2.2-5.8) | <0.001 | 3.3 (1.8-6.1) | <0.001 |
| Pleural effusion^a^* | 133 (61.0) | 40 (18.4) | 45 (20.6) | 1.7 (1.1-2.7) | 0.025 |  |  |
| **Treatment** |  |  |  |  |  |  |  |
| Discordant therapy | 4 (66.7) | 2 (33.3) | 0 (0.0) |  |  |  |  |
| Penicillin resistance^b^ | 61 (59.8) | 16 (15.7) | 25 (24.5) | 1.6 (0.9-2.9) | 0.110 |  |  |
| MDR^b^ | 9 (56.2) | 5 (31.3) | 2 (12.5) | 0.6 (0.2-1.9) | 0.430 |  |  |

Data are presented as No. (%) unless otherwise specified.

Abbreviations: IPD, invasive pneumococcal disease; CHD, chronic heart disease; COPD, chronic obstructive pulmonary disease; HIV, human immunodeficiency virus; MDR, multidrug resistance.

a Chest X-ray available for 488 adults during admission.

b MIC available for 310 adults.

*Variables tested on multivariate analysis
