# Supplementary material for: Risk factor profiles and clinical outcomes for children and adults with pneumococcal infections in Singapore: A need to expand vaccination policy?
Source: PLoS One. 2019 Oct 16;14(10):e0220951. doi: 10.1371/journal.pone.0220951 (PMC6795432; doi:10.1371/journal.pone.0220951)
Supplement: S3 Table — (DOCX) [file pone.0220951.s004.docx]

Supplementary Table 3. Factors associated with disease outcome at discharge in children with invasive *S. pneumoniae* infection.

|  | **Cured (%)**  **n=236** | **Discharged w/ sequelae (%) n=18** | **Death (%)**  **n=9** | **OR, univariate (95% CI)** | **P-value** | **OR, multivariate (95% CI)** | **P-value** |
| --- | --- | --- | --- | --- | --- | --- | --- |
| **Gender** |  |  |  |  |  |  |  |
| Male | 149 (93.7) | 7 (4.4) | 3 (1.9) | 1.0 |  |  |  |
| Female* | 87 (83.7) | 11 (10.6) | 6 (5.8) | 3.2 (0.8-13.0) | 0.107 |  |  |
| **Type of IPD** |  |  |  |  |  |  |  |
| Bacteremic pneumonia | 134 (92.4) | 7 (4.8) | 4 (2.8) | 1 |  |  |  |
| Bacteremia | 43 (97.7) | 1 (2.3) | 0 (0) |  |  |  |  |
| Meningitis* | 13 (52) | 7 (28) | 5 (20) | 8.8 (2.2-35.6) | 0.002 | 12.9 (1.2-139.2) | 0.035 |
| Others IPD | 16 (94.1) | 1 (5.6) | 0 (0) |  |  |  |  |
| Pneumonia | 30 (93.8) | 2 (6.3) | 0 (0) |  |  |  |  |
| **Comorbidities** |  |  |  |  |  |  |  |
| Any | 38 (82.6) | 5 (10.9) | 3 (6.5) | 2.5 (0.6-10.2) | 0.217 |  |  |
| CHD | 1 (0) | 0 (0) | 0 (0) |  |  |  |  |
| Asthma | 16 (88.9) | 2 (11.1) | 0 (0) |  |  |  |  |
| Immunocompromised | 18 (100) | 0 (0) | 0 (0) |  |  |  |  |
| HIV | 0 (0) | 0 (0) | 0 (0) |  |  |  |  |
| Chronic liver disease | 0 (0) | 0 (0) | 1 (100) |  |  |  |  |
| **Clinical** |  |  |  |  |  |  |  |
| Fever | 221 (89.5) | 18 (7.3) | 8 (3.2) | 0.5 (0.1-4.3) | 0.529 |  |  |
| Chest pain | 20 (100) | 0 (0) | 0 (0) |  |  |  |  |
| Acute cardiac events* | 1 (25) | 0 (0) | 3 (75) | 126.5 (11.4-1399.5) | <0.001 |  |  |
| Critical illness (PBS>4)* | 7 (35.0) | 6 (30) | 7 (35) | 64.9 (12.2-343.9) | <0.001 | 15.7 (2.4-102.2) | 0.004 |
| Bilateral Infiltrates^a^* | 23 (71.9) | 4 (12.5) | 5 (15.6) | 8.8 (2.2-35.0) | 0.002 | 9.4 (0.9-101.4) | 0.064 |
| Pleural effusion^a^ | 90 (90) | 7 (7) | 3 (3) | 0.5(0.1-2.3) | 0.359 |  |  |
| **Treatment** |  |  |  |  |  |  |  |
| Discordant therapy | 33 (0) | 0 (0) | 0 (0) |  |  |  |  |
| Penicillin resistance^b^ | 83 (93.3) | 5 (5.6) | 1 (1.1) | 0.3 (0.0-4.9) | 0.394 |  |  |
| MDR^b^ | 33 (89.2) | 4 (10.8) | 0 (0.0) | 1.2 (0.1-18.9) | 0.918 |  |  |

Data are presented as No. (%) unless otherwise specified.

Abbreviations: IPD, invasive pneumococcal disease; CHD, chronic heart disease; COPD, chronic obstructive pulmonary disease; HIV, human immunodeficiency virus; MDR, multidrug resistance.

a Chest X-ray available for 227 children during admission.

b MIC available for 125 children.

*Variables tested on multivariate analysis.
