# Supplementary material for: Risk factor profiles and clinical outcomes for children and adults with pneumococcal infections in Singapore: A need to expand vaccination policy?
Source: PLoS One. 2019 Oct 16;14(10):e0220951. doi: 10.1371/journal.pone.0220951 (PMC6795432; doi:10.1371/journal.pone.0220951)
Supplement: S4 Table — (DOCX) [file pone.0220951.s005.docx]

**Supplementary Table 4. Distribution of pneumococcal serotypes and clinical presentation in adults.**

| **Pneumococcal serotypes** | **Total no. of isolates** | **Bacteremic Pneumonia** | **Bacteremia** | **Meningitis** | **Others IPD** | **Pneumonia** | **Others non IPD** |
| --- | --- | --- | --- | --- | --- | --- | --- |
| **PCV-7** |  |  |  |  |  |  |  |
| 4 | 31 (5.5) | 24 (6.2) | 6 (7.5) | 1 (2.8) | 0 (0) | 0 (0) | 0 (0) |
| 6B | 44 (7.8) | 26 (6.7) | 11 (13.7) | 4 (11.1) | 0 (0) | 3 (10.3) | 0 (0) |
| 14 | 46 (8.2) | 38 (9.8) | 4 (5) | 1 (2.8) | 2 (9.5) | 1 (3.5) | 0 (0) |
| 18C | 1(0.2) | 1 (0.3) | 0 (0) | 0 (0) | 0 (0) | 0 (0) | 0 (0) |
| 19F | 32 (5.7) | 15 (3.9) | 6 (7.5) | 1 (2.8) | 4 (19.1) | 5 (17.2) | 1 (16.7) |
| 23F | 51 (9.1) | 34 (8.7) | 7 (8.7) | 4 (11.1) | 4 (19.1) | 1 (3.5) | 1 (16.7) |
| **PCV-10** |  |  |  |  |  |  |  |
| 1 | 14 (2.5) | 10 (2.6) | 0 (0) | 4 (11.1) | 0 (0) | 0 (0) | 0 (0) |
| 5 | 4 (0.7) | 4 (1.0) | 0 (0) | 0 (0) | 0 (0) | 0 (0) | 0 (0) |
| 7F | 24 (4.3) | 22 (5.7) | 0 (0) | 2 (5.6) | 0 (0) | 0 (0) | 0 (0) |
| **PCV-13** |  |  |  |  |  |  |  |
| 3 | 63 (11.2) | 49 (12.6) | 4 (5.0) | 3 (8.3) | 3 (14.3) | 3 (10.3) | 1 (16.7) |
| 6A | 5 (0.9) | 1 (0.3) | 2 (2.5) | 2 (5.6) | 0 (0) | 0 (0) | 0 (0) |
| 19A | 29 (5.2) | 25 (6.4) | 2 (2.5) | 0 (0.0) | 1 (4.8) | 1 (3.5) | 0 (0.0) |
| **PPV23** |  |  |  |  |  |  |  |
| 8 | 28 (5) | 21 (5.4) | 4 (5.0) | 2 (5.6) | 1(4.8) | 0 (0) | 0 (0) |
| 10A | 3 (0.5) | 1 (0.3) | 1 (1.3) | 1 (2.8) | 0 (0) | 0 (0) | 0 (0) |
| 12F | 7 (1.3) | 5 (1.3) | 1 (1.3) | 1 (2.8) | 0 (0) | 0 (0) | 0 (0) |
| 15B | 4 (0.7) | 3 (0.8) | 0 (0) | 0 (0) | 0 (0) | 0 (0) | 1 (16.7) |
| 22F | 8 (1.4) | 7 (1.8) | 0 (0) | 0 (0) | 0 (0) | 1 (3.5) | 0 (0.0) |
| **Non-vaccine** |  |  |  |  |  |  |  |
| 0 | 1 (0.2) | 0 (0) | 0 (0) | 1 (2.8) | 0 (0) | 0 (0) | 0 (0) |
| 2 | 2 (0.4) | 2 (0.5) | 0 (0) | 0 (0) | 0 (0) | 0 (0) | 0 (0) |
| 6 | 1 (0.2) | 1 (0.3) | 0 (0) | 0 (0) | 0 (0) | 0 (0) | 0 (0) |
| 6C | 14 (2.5) | 7 (1.8) | 5 (6) | 2 (5.6) | 0 (0) | 0 (0) | 0 (0) |
| 7A | 8 (1.4) | 7 (1.8) | 1 (0.3) | 0 (0) | 0 (0) | 0 (0) | 0 (0) |
| 7B | 1 (0.2) | 1 (0.3) | 0 (0) | 0 (0) | 0 (0) | 0 (0) | 0 (0) |
| 7C | 1 (0.2) | 0 (0) | 1 (1.3) | 0 (0) | 0 (0) | 0 (0) | 0 (0) |
| 9A | 2 (0.4) | 0 (0) | 1 (1.3) | 1 (2.8) | 0 (0) | 0 (0) | 0 (0) |
| 9L | 1 (0.2) | 0 (0) | 0 (0) | 0 (0) | 0 (0) | 1 (3.5) | 0 (0) |
| 11C | 1 (0.2) | 0 (0) | 1 (1.3) | 0 (0) | 0 (0) | 0 (0) | 0 (0) |
| 11D | 2 (0.4) | 2 (0.5) | 0 (0) | 0 (0) | 0 (0) | 0 (0) | 0 (0) |
| 13 | 2 (0.4) | 2 (0.5) | 0 (0) | 0 (0) | 0 (0) | 0 (0) | 0 (0) |
| 15 | 1 (0.2) | 0 (0) | 0 (0) | 1 (2.8) | 0 (0) | 0 (0) | 0 (0) |
| 15A | 10 (1.8) | 5 (1.3) | 1 (1.3) | 0 (0) | 1 (4.8) | 2 (6.9) | 1 (16.7) |
| 15C | 3 (0.5) | 1 (0.3) | 1 (1.3) | 0 (0) | 1 (4.8) | 0 (0) | 0 (0) |
| 15F | 8 (1.4) | 7 (1.8) | 0 (0) | 1 (2.8) | 0 (0) | 0 (0) | 0 (0) |
| 16F | 2 (0.4) | 2 (0.5) | 0 (0) | 0 (0) | 0 (0) | 0 (0) | 0 (0) |
| 17A | 2 (0.4) | 1 (0.3) | 0 (0) | 1 (2.8) | 0 (0) | 0 (0) | 0 (0) |
| 17F | 2 (0.4) | 1 (0.3) | 1 (1.3) | 0 (0) | 0 (0) | 0 (0) | 0 (0) |
| 18A | 1 (0.2) | 1 (0.3) | 0 (0) | 0 (0) | 0 (0) | 0 (0) | 0 (0) |
| 18B | 7 (1.3) | 4 (1) | 0 (0) | 0 (0) | 1 (4.8) | 0 (0) | 0 (0) |
| 18F | 3 (0.5) | 3 (0.8) | 0 (0) | 0 (0) | 0 (0) | 0 (0) | 0 (0) |
| 19B | 1 (0.2) | 1 (0.3) | 0 (0) | 0 (0) | 0 (0) | 0 (0) | 0 (0) |
| 20 | 10 (1.8) | 7 (1.8) | 3 (3.8) | 0 (0) | 0 (0) | 0 (0) | 0 (0) |
| 23A | 11 (2.0) | 5 (1.3) | 5 (6.3) | 0 (0) | 0 (0) | 1 (3.5) | 0 (0) |
| 23B | 1 (0.2) | 1 (0.3) | 0 (0) | 0 (0) | 0 (0) | 0 (0) | 0 (0) |
| 24A | 1 (0.2) | 1 (0.3) | 0 (0) | 0 (0) | 0 (0) | 0 (0) | 0 (0) |
| 25F | 5 (0.9) | 5 (1.3) | 0 (0) | 0 (0) | 0 (0) | 0 (0) | 0 (0) |
| 28A | 1 (0.2) | 0 (0) | 0 (0) | 0 (0) | 0 (0) | 1 (3.5) | 0 (0) |
| 28F | 2 (0.4) | 1 (0.3) | 0 (0) | 0 (0) | 0 (0) | 1 (3.5 | 0 (0) |
| 29 | 4 (0.7) | 2 (0.5) | 1 (1.3) | 0 (0) | 0 (0) | 1 (3.5) | 0 (0) |
| 31 | 1 (0.2) | 1 (0.3) | 0 (0) | 0 (0) | 0 (0) | 0 (0) | 0 (0) |
| 32A | 1 (0.2) | 0 (0) | 1 (1.3) | 0 (0) | 0 (0) | 0 (0) | 0 (0) |
| 32F | 1 (0.2) | 0 (0) | 0 (0) | 0 (0) | 0 (0) | 0 (0) | 1 (16.7) |
| 33B | 2 (0.40 | 1 (0.3) | 1 (1.3) | 0 (0) | 0 (0) | 0 (0) | 0 (0) |
| 34 | 5 (0.9) | 1 (0.3) | 3 (3.8) | 1 (2.8) | 0 (0) | 0 (0) | 0 (0) |
| 35B | 3 (0.5) | 1 (0.3) | 1 (1.3) | 1 (2.8) | 0 (0) | 0 (0) | 0 (0) |
| 35F | 1 (0.2) | 1 (0.3) | 0 (0) | 0 (0) | 0 (0) | 0 (0) | 0 (0) |
| 36 | 2 (0.4) | 1 (0.3) | 0 (0) | 0 (0) | 0 (0) | 1 (3.5) | 0 (0) |
| 38 | 4 (0.7) | 1 (0.30 | 2 (2.5) | 1 (2.8) | 0 (0) | 0 (0) | 0 (0) |
| 39 | 1 (0.2) | 0 (0) | 0 (0) | 0 (0) | 0 (0) | 1 (3.5) | 0 (0) |
| 44 | 1 (0.20 | 1 (0.30 | 0 (0) | 0 (0) | 0 (0) | 0 (0) | 0 (0) |
| 48 | 3 (0.5) | 2 (0.5) | 0 (0) | 0 (0) | 1 (4.8) | 0 (0) | 0 (0) |
| **Indeterminate** | 31 (5.5) | 23 (5.9) | 2 (2.5) | 0 (0) | 1 (4.8) | 5 (17.2) | 0 (0) |
