# Supplementary material for: Risk factor profiles and clinical outcomes for children and adults with pneumococcal infections in Singapore: A need to expand vaccination policy?
Source: PLoS One. 2019 Oct 16;14(10):e0220951. doi: 10.1371/journal.pone.0220951 (PMC6795432; doi:10.1371/journal.pone.0220951)
Supplement: S5 Table — (DOCX) [file pone.0220951.s006.docx]

**Supplementary Table 5. Distribution of pneumococcal serotypes and clinical presentation in children.**

| **Pneumococcal serotypes** | **Total no. of isolates** | **Bacteremic Pneumonia** | **Bacteremia** | **Meningitis** | **Others IPD** | **Pneumonia** | **Others non IPD** |
| --- | --- | --- | --- | --- | --- | --- | --- |
| **PCV-7** |  |  |  |  |  |  |  |
| 4 | 1 (0.3) | 1 (0.7) | 0 (0) | 0 (0) | 0 (0) | 0 (0) | 0 (0) |
| 6B | 65 (19.8) | 36 (24.8) | 9 (20.5) | 6 (24) | 4 (23.5) | 9 (13) | 1 (3.6) |
| 14 | 73 (22.3) | 38 (26.2) | 10 (22.7) | 6 (24) | 0 (0) | 14 (20.3) | 5 (17.9) |
| 18C | 1 (0.30 | 0 (0) | 0 (0) | 0 (0) | 0 (0) | 1 (1.5) | 0 (0) |
| 19F | 56 (17.1) | 10 (6.9) | 4 (9.1) | 5 (20) | 7 (41.2) | 19 (27.5) | 11 (39.3) |
| 23F | 26 (7.9) | 8 (5.5) | 9 (20.5) | 1 (4) | 1 (5.9) | 3 (4.4) | 4 (14.3) |
| **PCV-10** |  |  |  |  |  |  |  |
| 1 | 2 (0.6) | 2 (1.40 | 0 (0) | 0 (0) | 0 (0) | 0 (0) | 0 (0) |
| 5 | 2 (0.6) | 1 (0.7) | 1 (2.3) | 0 (0) | 0 (0) | 0 (0) | 0 (0) |
| 7F | 0 (0) |  |  |  |  |  |  |
| **PCV-13** |  |  |  |  |  |  |  |
| 3 | 8 (2.4) | 3 (2.1) | 0 (0) | 0 (0) | 1 (5.9) | 3 (4.4) | 1 (3.6) |
| 6A | 13 (4) | 7 (4.8) | 3 (6.8) | 1 (4) | 1 (5.9) | 1 (1.5) | 0 (0) |
| 19A | 43 (13.1) | 28 (19.3) | 3 (6.8) | 1 (4) | 0 (0) | 10 (14.5) | 1 (3.6) |
| **Non-vaccine** |  |  |  |  |  |  |  |
| 6C | 3 (0.9) | 1 (0.7) | 0 (0) | 1 (4) | 0 (0) | 1 (1.5) | 0 (0) |
| 6E | 1 (0.3) | 1 (0.7) | 0 (0) | 0 (0) | 0 (0) | 0 (0) | 0 (0) |
| 9A | 1 (0.3) | 1 (0.7) | 0 (0) | 0 (0) | 0 (0) | 0 (0) | 0 (0) |
| 11D | 2 (0.6) | 0 (0) | 1 (2.3) | 0 (0) | 0 (0) | 0 (0) | 1 (3.6) |
| 15A | 4 (1.2) | 0 (0) | 1 (2.3) | 0 (0) | 0 (0) | 2 (2.9) | 1 (3.6) |
| 15B | 1 (0.3) | 0 (0) | 0 (0) | 1 (4) | 0 (0) | 0 (0) | 0 (0) |
| 15C | 4 (1.2) | 0 (0) | 1 (2.3) | 1 (4) | 1 (5.9) | 1 (1.5) | 0 (0) |
| 18B | 1 (0.3) | 1 (0.7) | 0 (0) | 0 (0) | 0 (0) | 0 (0) | 0 (0) |
| 20 | 1 (0.3) | 0 (0) | 0 (0) | 0 (0) | 0 (0) | 1 (1.5) | 0 (0) |
| 23A | 2 (0.6) | 1 (0.7) | 1 (2.3) | 0 (0) | 0 (0) | 0 (0) | 0 (0) |
| 33F | 1 (0.3) | 1 (0.7) | 0 (0) | 0 (0) | 0 (0) | 0 (0) | 0 (0) |
| 34 | 1 (0.3) | 0 (0) | 0 (0) | 0 (0) | 0 (0) | 0 (0) | 1 (3.6) |
| **Indeterminate** | 16 (4.9) | 5 (3.5) | 1 (2.3) | 2 (8) | 2 (11.8) | 4 (5.8) | 2 (7.1) |
