# Supplementary material for: Risk factor profiles and clinical outcomes for children and adults with pneumococcal infections in Singapore: A need to expand vaccination policy?
Source: PLoS One. 2019 Oct 16;14(10):e0220951. doi: 10.1371/journal.pone.0220951 (PMC6795432; doi:10.1371/journal.pone.0220951)
Supplement: S6 Table — (DOCX) [file pone.0220951.s007.docx]

**Supplementary Table 6. Changes in proportion of Adults PD serotypes from period 1 to period 2.**

| **Pneumococcal serotypes** | **Period 1, n (%)** | **Period 2, n (%)** | |
| --- | --- | --- | --- |
|  |  | **Early period (2005-2009)** | **Late period (2010-1013)** |
| 3 | 9 (14.3) | 22 (9.2) | 32 (12.4) |
| 23F | 3 (4.8) | 31 (13.0) | 17 (6.6) |
| 14 | 7 (11.1) | 26 (10.8) | 13 (5.0) |
| 6B | 7 (11.1) | 13 (5.4) | 24 (9.3) |
| 19F | 4 (6.4) | 15 (6.3) | 13 (5.0) |
| 4 | 5 (8.0) | 14 (5.8) | 12 (4.7) |
| 19A | 1 (1.6) | 8 (3.3) | 20 (7.8) |
| 8 | 0 (0.0) | 18 (7.5) | 10 (3.9) |
| Others^a^ | 27 (42.9) | 93 (38.8) | 117 (45.4) |

Data are presented as No. (%) unless otherwise specified.

Abbreviations: PD, pneumococcal disease

^a^ Other serotypes (detected <5%) included 18C, 1, 2 5, 6, 6A, 6C, 7A, 7B, 7C, 7F, 9A, 9L, 10A, 11C, 11D, 12F, 13, 15, 15A, 15C, 17F, 16F, 17A, 17F, 18A, 18B, 18F, 19B, 20, 23A, 23B, 24A, 25F, 28A, 28F, 29, 31, 32A, 32F, 33B, 34, 35B, 37F.
