# Supplementary material for: Risk factor profiles and clinical outcomes for children and adults with pneumococcal infections in Singapore: A need to expand vaccination policy?
Source: PLoS One. 2019 Oct 16;14(10):e0220951. doi: 10.1371/journal.pone.0220951 (PMC6795432; doi:10.1371/journal.pone.0220951)
Supplement: S7 Table — (DOCX) [file pone.0220951.s008.docx]

**Supplementary Table 7. Changes in proportion of Children PD serotypes from period 1 to period 2.**

| **Pneumococcal serotypes** | **Period 1, n (%)** | **Period 2, n (%)** | |
| --- | --- | --- | --- |
|  |  | **Early period (2005-2009)** | **Late period (2010-1013)** |
| 14 | 40 (25.8) | 23 (22.8) | 10 (13.9) |
| 19F | 35 (22.6) | 11 (10.9) | 10 (13.9) |
| 6B | 27 (17.4) | 28 (27.7) | 10 (13.9) |
| 23F | 19 (12.3) | 6 (5.9) | 1 (1.4) |
| 19A | 7 (4.5) | 12 (11.9) | 24 (33.3) |
| Others^a^ | 27 (17.4) | 21 (20.8) | 17 (23.6) |

Data are presented as No. (%) unless otherwise specified.

Abbreviations: PD, pneumococcal disease

^a^ Other serotypes (detected <5%) included 4, 18C, 1, 5, 3, 6A, 6C, 6E, 9A, 11D, 15A, 15B, 15C, 18B, 20, 23A, 1, 34.
