# Supplementary material for: Risk factor profiles and clinical outcomes for children and adults with pneumococcal infections in Singapore: A need to expand vaccination policy?
Source: PLoS One. 2019 Oct 16;14(10):e0220951. doi: 10.1371/journal.pone.0220951 (PMC6795432; doi:10.1371/journal.pone.0220951)
Supplement: S1 Codebook — (DOC) [file pone.0220951.s010.doc]

**Pneumococcal Study Codebook**

| **Variable Name** | **Descriptions** | **Data Type** | **Coding** |
| --- | --- | --- | --- |
| Serotype | Isolate serotype | Text |  |
| ICU  To code if ‘SiteOfCare’=0 | Whether patient went to ICU. | Number | 0=No  1=Yes  2=Unknown |
| Gender | Gender | Number | 0=Male 1=Female |
| NosoComplic | Is this nosocomial disease? (defined as isolation >3 days from admission) | Number | 0=No  1=Yes  2=Unknown |
| viralcoinfection | Is there viral co-infection? | Number | 0=No  1=Yes |
| LivingCondition | Computed from LivingSituation | Number | 0=At home  1=In nursing home  2= Others (shelter, military base, prision…)  3=unknown |
| Ethnic | Ethnicity | Number | 0= Chinese 1= Malay  2= Indian  3= Others |
| CHD | Chronic heart disease (exclude hypertension) | Number | 0=No  1=Yes  2=Unknown |
| ChronicLiverDisease | Chronic liver disease (including cirrhosis) | Number | 0=No  1=Yes  2=Unknown |
| DM | Diabetes Mellitus | Number | 0=No  1=Yes  2=Unknown |
| Alcoholism | Alcoholism | Number | 0=No  1=Yes  2=Unknown |
| SMK | Cigarette smoking | Number | 0=No  1=Yes  2=Unknown |
| Dementia | Dementia | Number | 0=No  1=Yes  2=Unknown |
| PenumoVac | Has the patient received pneumococcal vaccination? | Number | 0= No  1= Yes  2= Unknown |
| MechVentilation | Mechanical ventilation | Text | a-No  b-Yes  c=Unknown |
| CXRPe  To code if ‘CXRNormal’=0 | Pleural effusion on CXR | Number | 0= No  1= Yes  2=Unknown |
| O2 | Supplemental oxygen | Number | 0=No  1=Yes  2=Unknown |
| NonIMV | Non-invasive mechanical ventilation (e.g. BIPAP, CPAP) | Number | 0=No  1=Yes  2=Unknown |
| IMV | Invasive mechanical ventilation | Number | 0=No  1=Yes  2=Unknown |
| PBS | PBS score | Number | 0=No  1=Yes  2=Unknown |
| RRT | Renal replacement therapy or dialysis | Number | 0=No  1=Yes  2=Unknown |
| InotropVasop | Did the patients receive inotropes and/or vasopressors? | Number | 0=No  1=Yes  2=Unknown |
| Blood | Did patient receive blood transfusion or blood products? | Number | 0=No  1=Yes  2=Unknown |
| Sx | Did patient go under surgical procedures? | Number | 0=No  1=Yes  2=Unknown |
| AcLungInjuryARDS | Acute lung injury/ Acute respiratory distress syndrome | Number | 0=No  1=Yes  2=Unknown |
| Fever | Has the patient history of fever (>38°C)? | Number | 0=No  1=Yes  2=Unknown |
| ChestPain | Chest pain | Number | 0=No  1=Yes  2=Unknown |
| Seizures | Seizures | Number | 0=No  1=Yes  2=Unknown |
| Pneumothorax | Pneumothorax | Number | 0=No  1=Yes  2=Unknown |
| Seizure | Seizure(s) | Number | 0=No  1=Yes  2=Unknown |
| Stroke | Stroke | Number | 0=No  1=Yes  2=Unknown |
| CoagulopathyDIC | Coagulopathy or Disseminated intravascular coagulation | Number | 0=No  1=Yes  2=Unknown |
| RhabdoMyositis | Rhabdomyolysis or myositis | Number | 0=No  1=Yes  2=Unknown |
| ARF | Acute renal injury/failure | Number | 0=No  1=Yes  2=Unknown |
| GIBleeding | Gastrointestinal bleeding | Number | 0=No  1=Yes  2=Unknown |
| Hyperglycemia | Hyperglycemia | Number | 0=No  1=Yes  2=Unknown |
| Hypoglycemia | Hypoglycemia | Number | 0=No  1=Yes  2=Unknown |
| Death | Did the patient die in the hospital? | Number | 0=No  1=Yes  2= Unknown |
| SelfCareDischarge  To code if ‘Death’=0 | Is the patient able to self-care at discharge versus prior illness? | Number | 0=Same as prior illness  1=Decreased  2= Increased  3= Unknown |
| Age | Age | Number |  |
| LOS | Length of stay in the hospital | Number | Free text |
| AtLeast1_Comorbidity | The patient presents at least 1 comorbidity | Number | 0=No  1=Yes |
| Asthma | Asthma | Number | 0=No  1=Yes |
| COPD | COPD | Number | 0=No  1=Yes |
| Immunocomp | All immunocompromised patients except those with HIV | Number | 0=No  1=Yes |
| HIV | HIV patients | Number | 0=No  1=Yes |
| ACardiacEv | Patient with MI or CHF or Arrhytmia | Number | 0=No  1=Yes |
| Renal insufficiency | Renal insufficiency | Number | 0=No  1=Yes |
| selfcaredischarge | Is the patient able to self-care at discharge versus prior illness? | Number | 0=Cured  1=Discharged with sequelae  2= Death |
| time2die | Number of days from admission to death | Number | Text |
| Bi_Inflitrates | Bilateral infiltrates in CXR | Number | 0=No  1=Yes |
| timead2abx | Number of days from admission to start antibiotics | Number | Free text |
| Clin_Synd_Others_Short | Clinical Syndromes presented | Number | 0=Bacteremic pneumonia  1=Bacteraemia without focus  2=Meningitis  3=Others IPD  4=Pneumonia  5=Others non IPD |
| Tetracycline (S/NS) | Tetracycline sensitivity following CLSI document M100-S24, 2014) | Number | 0=Susceptible  1=Resistant |
| Ceftriaxone (S/NS) | Ceftriaxone sensitivity following CLSI document M100-S24, 2014) | Number | 0=Susceptible  1=Resistant |
| Penicillin (S/NS) | Penicillin sensitivity following CLSI document M100-S24, 2014) | Number | 0=Susceptible  1=Resistant |
| Erythromycin (S/NS) | Erythromycin sensitivity following CLSI document M100-S24, 2014) | Number | 0=Susceptible  1=Resistant |
| MDR | Multidrug resistance: an isolate resistant to ≥3 antibiotics | Number | 0=No  1=Yes |
| Concordant_therapy | Defined as receipt within the first 48 hours of treatment >= antibiotic to witch a pneumococcal isolate was susceptible | Number | 0=No  1=Yes |
